# Prediction of response to cardiac resynchronization therapy using left ventricular pacing lead position and cardiovascular magnetic resonance derived wall motion patterns: a prospective cohort study

**DOI:** 10.1186/s12968-015-0158-5

**Published:** 2015-07-14

**Authors:** Gregory R. Hartlage, Jonathan D. Suever, Stephanie Clement-Guinaudeau, Patrick T. Strickland, Nima Ghasemzadeh, R. Patrick Magrath, Ankit Parikh, Stamatios Lerakis, Michael H. Hoskins, Angel R. Leon, Michael S. Lloyd, John N. Oshinski

**Affiliations:** Department of Radiology and Imaging Sciences, Emory University School of Medicine, Atlanta, GA USA; Department of Medicine, Division of Cardiology, Emory University School of Medicine, Atlanta, GA USA; Department of Biomedical Engineering, Georgia Institute of Technology/Emory University, Atlanta, GA USA

**Keywords:** Cardiovascular magnetic resonance, Dyssynchrony, Cardiac resynchronization therapy

## Abstract

**Background:**

Despite marked benefits in many heart failure patients, a considerable proportion of patients treated with cardiac resynchronization therapy (CRT) fail to respond appropriately. Recently, a “U-shaped” (*type II*) wall motion pattern identified by cardiovascular magnetic resonance (CMR) has been associated with improved CRT response compared to a homogenous (*type I*) wall motion pattern. There is also evidence that a left ventricular (LV) lead localized to the latest contracting LV site predicts superior response, compared to an LV lead localized remotely from the latest contracting LV site.

**Methods:**

We prospectively evaluated patients undergoing CRT with pre-procedural CMR to determine the presence of *type I* and *type II* wall motion patterns and pre-procedural echocardiography to determine end systolic volume (ESV). We assessed the final LV lead position on post-procedural fluoroscopic images to determine whether the lead was positioned concordant to or remote from the latest contracting LV site. CRT response was defined as a ≥ 15 % reduction in ESV on a 6 month follow-up echocardiogram.

**Results:**

The study included 33 patients meeting conventional indications for CRT with a mean New York Heart Association class of 2.8 ± 0.4 and mean LV ejection fraction of 28 ± 9 %. Overall, 55 % of patients were echocardiographic responders by ESV criteria. Patients with both a *type II* pattern and an LV lead concordant to the latest contracting site (T2CL) had a response rate of 92 %, compared to a response rate of 33 % for those without T2CL (*p* = 0.003). T2CL was the only independent predictor of response on multivariate analysis (odds ratio 18, 95 % confidence interval 1.6-206; *p* = 0.018). T2CL resulted in significant incremental improvement in prediction of echocardiographic response (increase in the area under the receiver operator curve from 0.69 to 0.84; *p* = 0.038).

**Conclusions:**

The presence of a *type II* wall motion pattern on CMR and a concordant LV lead predicts superior CRT response. Improving patient selection by evaluating wall motion pattern and targeting LV lead placement may ultimately improve the response rate to CRT.

## Background

Cardiac resynchronization therapy (CRT) improves morbidity and mortality in many heart failure patients, yet 1 in 3 patients fail to respond [1]. The pursuit of echocardiographic measures of myocardial dyssynchrony to refine patient selection has been disappointing [2], and current CRT selection criteria include only functional class, left ventricular (LV) ejection fraction (EF), QRS duration, and QRS morphology [3]. In general, favorable electrocardiographic criteria associated with improved response are a left bundle branch block (LBBB) morphology and a QRS duration greater than 150 ms [4–6]. To better refine these clinical criteria, an evolving understanding of the electrical and mechanical substrate within the myocardium is needed [7–15].

A “U-shaped” (*type II*) LV wall motion pattern, suggestive of electrical conduction block, can be demonstrated with cardiovascular magnetic resonance (CMR), and has been associated with a superior CRT response compared to a more homogenous (*type I*) pattern [12]. Other studies have demonstrated that targeting the LV pacing lead to the latest site of LV contraction improves patient response [8–11]. Both strain echocardiography and CMR can identify the latest contracting LV site and areas of non-viable scar unsuitable for pacing [8–11, 16, 17]. CRT guided by such methods has yielded results superior to conventional implantation in randomized controlled trials [8, 9].

To date, no studies have evaluated LV lead location relative to specific patterns of LV wall motion in patients undergoing CRT. We investigated how CMR-derived wall motion patterns interact with LV lead location to influence response to CRT. We hypothesized that patients with both a *type II* wall motion pattern and an LV lead located at the latest contracting site would have a superior CRT response compared to those with only one or neither of these characteristics.

## Methods

### Patient selection

From 2003 to 2013, we prospectively recruited consecutive patients being referred for CRT. All patients had systolic heart failure (EF ≤ 35 % by transthoracic echocardiography), QRS duration > 120 ms, and New York Heart Association functional class II or III symptoms despite optimal medical therapy. Patients were enrolled only if they would be able to follow up 6 months after the CRT procedure and if they had no known contraindications to CMR. The Emory University institutional review board approved the study and all patients gave written informed consent prior to enrollment.

### Electrocardiogram classification

A favorable electrocardiogram (ECG) was defined as true LBBB morphology and QRS duration > 150 ms. True LBBB morphology was classified as a QS or rS complex in V1 and/or V2; monophasic R wave in leads I, aVL, V5, and V6; and mid QRS notching or slurring in at least two of the following leads: I, aVL, V1, V2, V5, or V6. Non-favorable ECGs were those that demonstrated an atypical LBBB, an intraventricular conduction delay not satisfying criteria for true LBBB, or a QRS duration < 150 ms. Given that significant intraventricular conduction delay may exist in the presence of right bundle branch block [14, 18], patients with bifascicular block patterns were included in the analysis, but those with isolated right bundle branch blocks were excluded.

### Transthoracic echocardiography

Patients underwent two-dimensional transthoracic echocardiography at baseline and at 6 month follow-up. The echocardiographic studies were performed on a General Electric Vivid 7 (Milwaukee, Wisconsin). LV end-systolic volume (ESV), end-diastolic volume, and EF were assessed by Simpson’s modified biplane method of discs using the apical four-chamber and apical two-chamber views. All echocardiograms were reviewed by a board-certified reader blinded to baseline and follow-up status.

### Cardiovascular magnetic resonance

CMR was performed on a 1.5 T Siemens Avanto scanner (Erlangen, Germany) with a 5-element phased array coil and ECG triggering. Steady-state free precession (SSFP) short-axis images were acquired parallel to the mitral valve plane to cover the entire length of the LV (8 mm slices with no slice gap). Two-, three-, and four-chamber cine images were also acquired. Late gadolinium enhancement (LGE) CMR was performed with a phase-sensitive inversion recovery sequence 10–15 minutes after the administration of 0.1 mmol/kg MultiHance (gadobenate dimeglumine; Bracco Diagnostics, Singen, Germany). LGE images were acquired in the basal, mid, and apical short axis, as well as the two-, three-, and four-chamber views. Significant scar was defined as enhancement in > 15 % of LV myocardium [19].

### Left ventricular wall motion analysis

Endocardial borders were traced on each frame of the short-axis cine images and radial displacement curves were generated as previously described [20]. Briefly, radial displacement curves were generated by measuring the radial distance of the endocardial contour relative to the LV centroid at 100 circumferentially spaced points for each slice. To account for translation of the LV over the cardiac cycle, the LV centroid was determined from the location of the mitral valve annulus and apex on every frame in the two and four-chamber views. Regional wall motion delay times were determined by cross-correlating each radial displacement curve to a patient-specific reference curve and recording the delay time for peak correlation. Regional radial displacement curves were compared visually to long and short axis cines for regional myocardial thickening and LGE images to determine akinetic segments with passive movement, which were excluded from wall motion analysis.

**Fig. 1:**
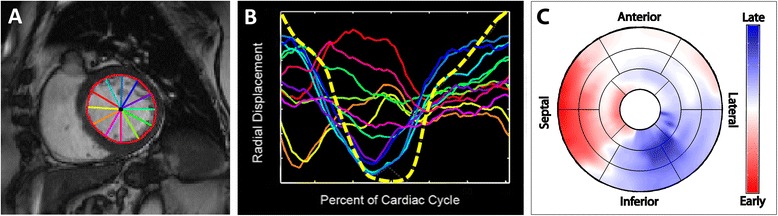
CMR processing and wall motion pattern analysis. Endocardial contours were traced on short-axis cine images (**a**; *red circle*) and the distance to the centroid computed for each site (**b**). Each color *curve* represents a corresponding colored line from **a**; 100 sites were sampled per slice, however, only 12 are shown here for graphical simplicity. Each regional radial displacement curve is compared by cross-correlation (sliding in time) to a patient-specific reference (**b**; *yellow dotted line*) to determine the mechanical delay time. Delay times are mapped to a modified American Heart Association 17-segment model (**c**). Note the early motion in the septal segments in **b**, shown in *red* in **c**, represents the septal flash

**Fig. 2:**
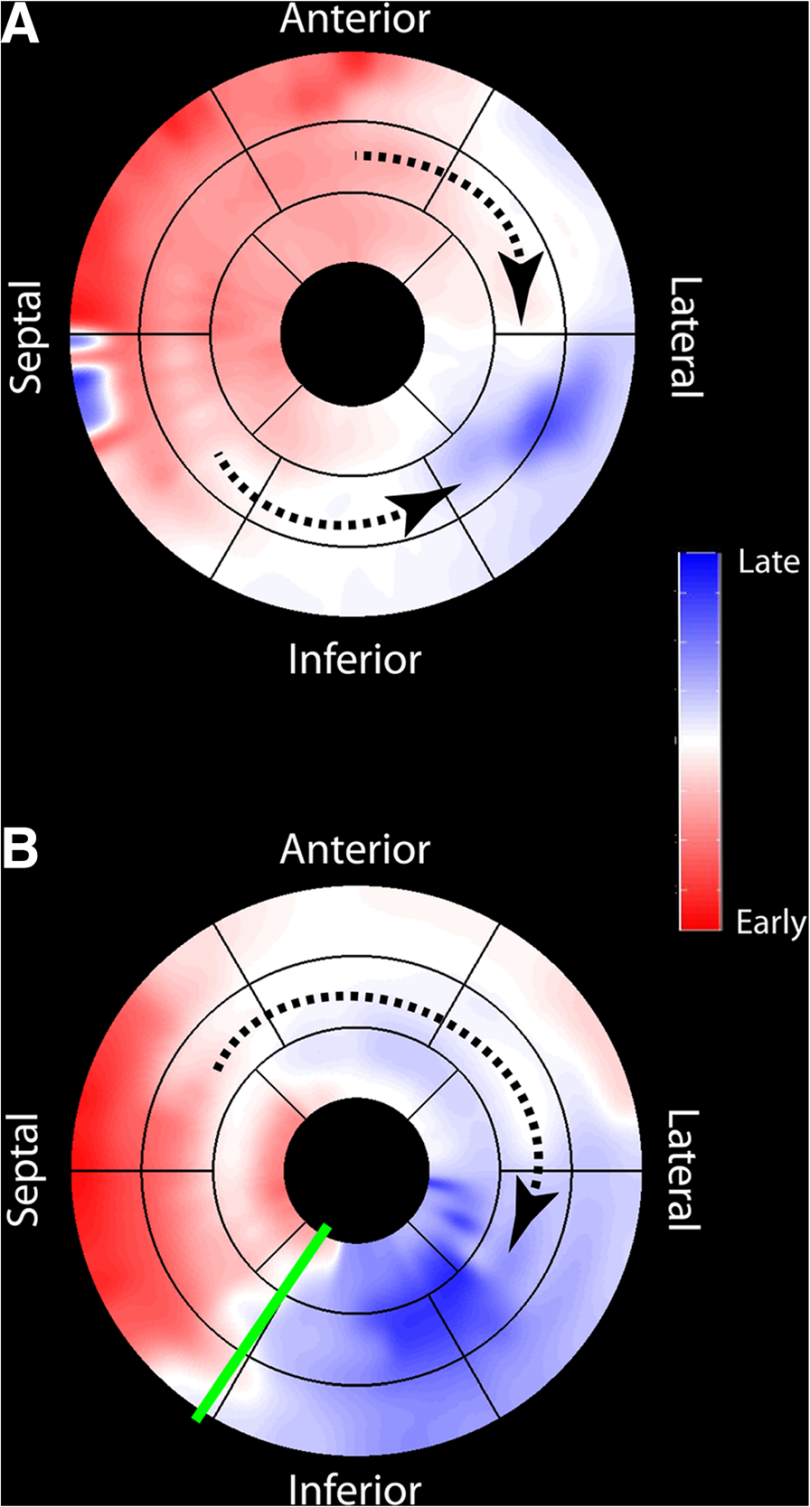
*Type I* and *type II* wall motion patterns. Modified American Heart Association models demonstrating (**a**) a *type I* wall motion pattern, with *dotted lines* indicating homogenous wave fronts anteriorly and inferiorly towards the lateral wall, and (**b**) a *type II* wall motion pattern, with an inferior line of block (*green line*) and *dotted line* indicating an anteriorly directed wave front and late inferior wall motion

Regional wall motion delays were determined throughout the LV (excluding the apex) and then mapped to a modified American Heart Association 17-segment model [21] (Fig. 1). LV wall motion patterns were categorized as *type I* if the wave front proceeded homogenously from the septum to the LV free wall (no adjacent early and late segments) and *type II* if the wave front was heterogeneous with evidence of an inferred line of block (adjacent early and late segments; Fig. 2). Septal flash was identified by rapid inward and outward motion during isovolumic contraction involving at least one of the septal segments. Isovolumic contraction time was characterized as the interval from the onset of LV contraction to aortic valve opening as visualized on long-axis cine SSFP images and confirmed by radial displacement curve analysis. In areas of septal flash, the time to peak radial displacement was defined as the initial radial displacement, rather than a subsequent peak likely representing rebound.

### Identification of the latest contracting site

The number of discrete myocardial sites sampled per segment ranged from 50–100, depending on the number of short axis slices obtained (i.e. the LV length) and the apical versus mid or basal position. The latest contracting site was defined as latest single site to reach maximum radial displacement. The segment containing this site was defined as the *latest contracting segment*.

### Coronary venography and device implantation

The implanting electrophysiologist had no knowledge of the CMR derived wall motion patterns prior to implantation. All CRT devices were implanted using standard procedure. Briefly, central venous access was gained via either the subclavian vein or cephalic vein cut-down. Biplane balloon catheter coronary venography defined coronary venous anatomy in the 30° right-anterior-oblique and 30° left-anterior-oblique views. A suitable anterolateral, lateral, or posterolateral coronary vein was selected based solely on anatomic characteristics; the CMR data was not used to guide this decision.

### Left ventricular lead localization

**Fig. 3:**
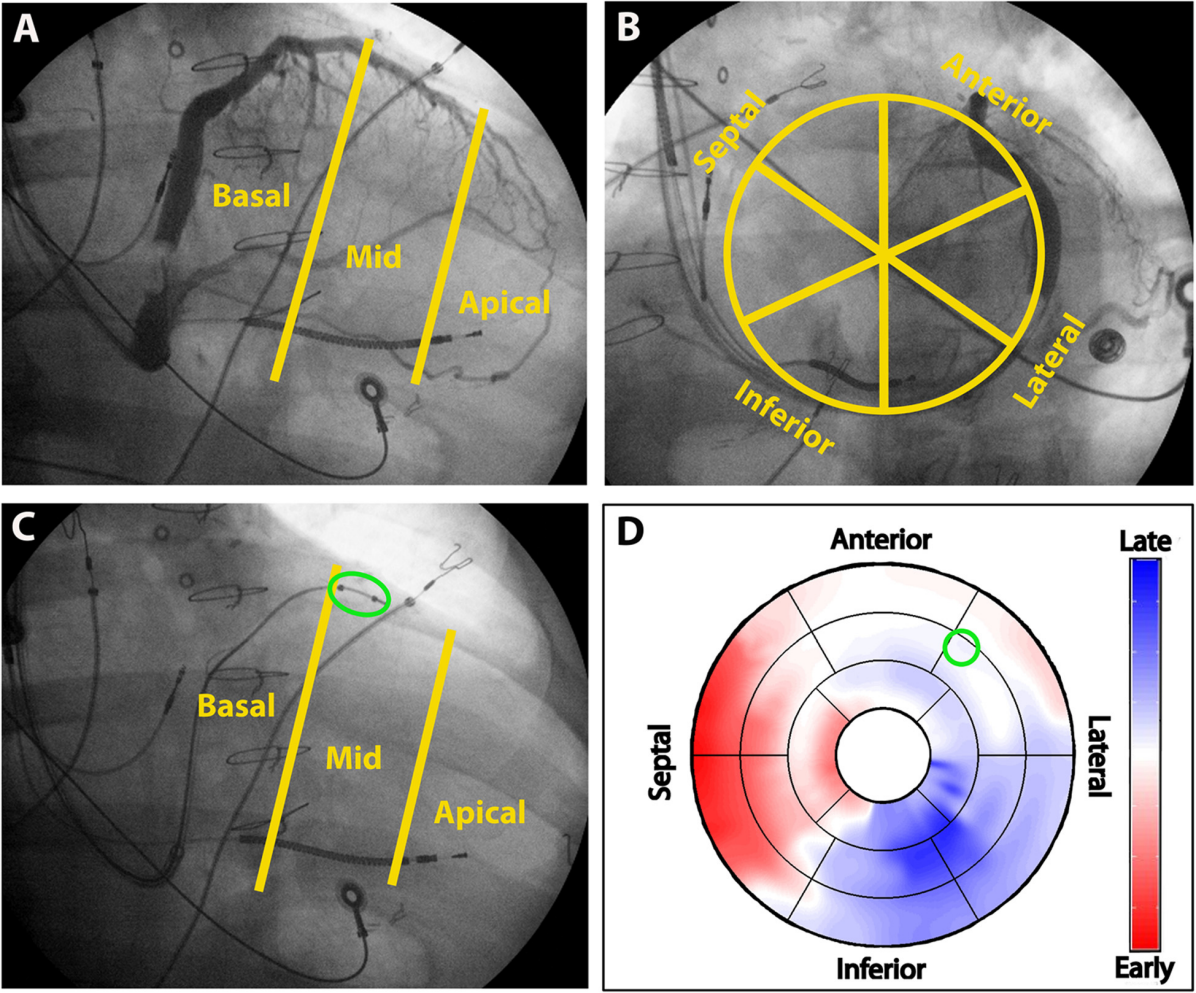
Left ventricular lead localization. Biplane venograms (**a**; *right anterior oblique 30°*, **b**: *left anterior oblique 30°*) and lead localizing still frame (**c**; *right anterior oblique 30°*, left anterior oblique 30° not shown) to map left ventricular pacing lead locations (*green ellipse* on **c**) onto the modified American Heart Association model (**d**; *green circle* denotes lead location). Left anterior oblique images were used to determine the circumferential location while right anterior oblique images were used for longitudinal position

Final LV lead position was determined by biplane fluoroscopy and comparison to baseline venous anatomy by coronary venography. The right anterior oblique image defined long-axis position, while the left anterior oblique image defined short-axis position (Fig. 3). Using these images, LV lead position was mapped to the modified American Heart Association 17-segment model. As described in previous studies [8, 9], final LV lead placement was considered *concordant* if placed in a viable segment within 1 segment of the latest contracting segment, and *remote* if placed more than 1 segment from the latest contracting segment. If the LV lead was placed in a non-viable segment with > 50 % transmural LGE, lead position was considered remote.

### Study outcome

The primary outcome was positive echocardiographic response to CRT, defined as reverse LV remodeling with a reduction in ESV by ≥ 15 % at 6 months. We compared response in patients with both a *type II* pattern and concordant lead (T2CL) to those without both of these findings (i.e. *type I* pattern and/or remote leads).

### Statistical analysis

Statistical analysis was performed with SPSS version 20 (Chicago, Illinois) and SAS version 9.3 (Cary, North Carolina). Dichotomous variables were expressed as numbers and percentages, while continuous variables were expressed as mean ± standard deviation. Fisher’s Exact test was used to compare categorical variables. Continuous variables were compared by the Student’s *t*-test (normal distribution) or the Mann–Whitney *U* test (non-normal distribution). Predictors of CRT response were analyzed by univariate binary logistic regression analysis. Independent predictors were analyzed by multivariate binary logistic regression analysis in a model including traditional variables associated with CRT response (favorable ECG and non-ischemic cardiomyopathy, NICM). A 1000 sample bootstrapping analysis was done to assess internal validity of the multivariate model using SPSS. In an exploratory analysis, novel imaging variables (presence of significant scar, maximum opposite wall delay, and septal flash on CMR) were added to the multivariate model to evaluate for an additional effect. The incremental benefit of T2CL was assessed by improvement in the area under the receiver-operating curve (AUC) before and after its addition to the multivariate model. Cohen’s kappa coefficient was used to test the interobserver variability of CMR wall motion pattern and LV lead position. Statistical significance was determined by *p* < 0.05 on two-tailed analysis.

## Results

### Patient characteristics

Thirty-three patients were included in our analysis. Thirteen patients (39 %) were determined to have a *type I* (homogeneous) wall motion pattern, while 20 (61 %) had a *type II* (U-shaped) wall motion pattern. Two of the patients had bifascicular block (6 %); both of these patients had *type I* rather than *type II* contraction patterns. *Type II* patterns were present in 85 % of patients with favorable ECGs and 45 % of patients with non-favorable ECGs. Of the 20 patients with a *type II* pattern, 15 (75 %) had an inferior line of block and 5 (25 %) had an anterior line of block. The latest contracting segment was adjacent to the line of block in 19 of the 20 patients (95 %).

Among the 33 patients in the cohort, the LV lead was concordant in 18 patients (54 %) and remote in 15 patients (46 %). True concordance, with the presence of the LV lead directly within the latest contracting segment, was observed in one patient; the remaining 17 (94 %) had the LV lead adjacent to the latest contracting segment. In 14 of these patients (82 %), the lead was in the next latest contracting segment. A full set of baseline characteristics for all patients and then grouped by echocardiographic response is shown in Table 1. Relevant baseline findings grouped by wall motion pattern is shown in Table 2. Patients with *type II* patterns were more likely to have a favorable ECG, less significant scar on LGE, septal flash, and longer maximum opposing wall delays compared to those with *type I* patterns.

**Table 1:** Patient characteristics

	All Patients	Responders	Non-responders	*P* value
	*N* = 33	*N* = 18 (55 %)	*N* = 15 (45 %)	
Age (years)	61 ± 13	61 ± 13	62 ± 12	0.72
Male, n (%)	18 (55)	7 (39)	11 (73)	0.08
NICM, n (%)	26 (79)	17 (94)	9 (60)	0.03
NHYA functional class	2.8 ± 0.4	2.8 ± 0.4	2.8 ± 0.4	0.86
Comorbidities, n (%)				
Coronary artery disease	10 (30)	2 (11)	8 (53)	0.02
Diabetes mellitus	9 (27)	5 (28)	4 (27)	0.94
Hypertension	16 (49)	8 (44)	8 (53)	0.73
Dyslipidemia	17 (52)	8 (44)	9 (60)	0.49
Chronic kidney disease	7 (21)	3 (17)	4 (27)	0.67
Atrial fibrillation	7 (21)	4 (22)	3 (20)	0.88
Medications, n (%)				
ACE inhibitor/ARB	32 (97)	18 (100)	14 (93)	0.46
Beta-blocker	32 (97)	18(100)	14 (93)	0.46
Aldosterone antagonist	11 (33)	6 (33)	5 (33)	1.00
Diuretic	23 (70)	13 (72)	10 (67)	0.73
Statin	18 (55)	8 (44)	10 (67)	0.30
Clinical variables				
Body mass index (kg/m^2^)	28 ± 5	29 ± 4	28 ± 6	0.89
Resting heart rate (bpm)	73 ± 14	75 ± 17	71 ± 10	0.42
Serum creatinine (mg/dl)	1.06 ± 0.24	1.06 ± 0.24	1.07 ± 0.26	0.90
Favorable ECG pattern^a^, n (%)	13 (39)	9 (50)	4 (27)	0.28
Echocardiographic variables				
End-systolic volume (ml)	150 ± 71	143 ± 61	159 ± 84	0.55
Ejection fraction (%)	28 ± 9	27 ± 8	30 ± 9	0.37
Mitral regurgitation grade	1.1 ± 0.8	1.1 ± 0.7	1.1 ± 0.9	0.87
CMR findings				
Maximum wall delay (ms)	432 ± 120	455 ± 104	404 ± 135	0.23
*Type II* pattern, n (%)	20 (61)	14 (78)	6 (40)	0.038
Septal flash, n (%)	27 (82)	17 (94)	10 (67)	0.07
No scar, n (%)	25 (76)	16 (89)	9 (60)	0.10
Concordant CRT lead, n (%)	18 (55)	13 (72)	5 (28)	0.038
Combined *type II* pattern and concordant CRT lead, n (%)	12 (36)	11 (61)	1 (7)	0.003

^a^A favorable ECG pattern is indicated by the presence of a true left bundle branch block and a QRS duration > 150 ms

Values are presented as n (%) or mean ± standard deviation. ACE = Angiotensinogen Converting Enzyme; ARB = Angiotensin Receptor Blocker; CMR = Cardiovascular Magnetic Resonance; CRT = Cardiac Resynchronization Therapy; ECG = Electrocardiogram; LBBB = Left Bundle Branch Block; NYHA = New York Heart Association; NICM = Non-Ischemic Cardiomyopathy

**Table 2:** CMR wall motion pattern associations

	*Type I*	*Type II*	*P* value
	*N* = 13 (39 %)	*N* = 20 (61 %)	
Etiology			
NICM, n (%)	8 (62)	18 (90)	0.084
ICM, n (%)	5 (38)	2 (10)	
ECG			
Favorable^a^, n (%)	2 (15)	11 (55)	0.032
Non-favorable, n (%)	11 (85)	9 (45)	
CMR			
Maximum wall delay (ms)	371 ± 117	471 ± 106	0.016
Late gadolinium enhancement			
Scar, n (%)	6 (46)	2 (10)	0.035
No scar, n (%)	7 (54)	18 (90)	
Septal flash			
Present, n (%)	7 (54)	20 (100)	0.002
Absent, n (%)	6 (46)	0 (0)	
CRT lead location			
Lead concordant, n (%)	6 (46)	12 (60)	0.49
Lead remote, n (%)	7 (54)	8 (40)	

^a^A favorable ECG pattern is indicated by the presence of a true left bundle branch block and a QRS duration > 150 ms

Values are presented as n (%) or mean ± standard deviation. CMR = Cardiovascular Magnetic Resonance; CRT = Cardiac Resynchronization Therapy; ECG = Electrocardiogram; ICM = Ischemic Cardiomyopathy; NICM = Non-Ischemic Cardiomyopathy

### Response: baseline clinical and imaging variables

The median time from CRT implantation to follow-up echocardiogram was 198 days (interquartile range 184 to 227 days). The overall echocardiographic response rate was 55 %. Responders were more likely to have NICM, a *type II* pattern, a concordant LV lead, and T2CL, as shown in Table 1. Otherwise, there were no significant differences between responders and non-responders.

### Response: wall motion patterns and lead position

The response rate for T2CL patients was significantly higher than non-T2CL patients, with intermediate response rates for all patients with a *type II* pattern regardless of lead location and all patients with a concordant LV lead position regardless of wall motion pattern (Table 3). Overall, all *type I* pattern patients had a 31 % response rate and all remote LV lead patients had a 33 % response rate.

**Table 3:** Echocardiographic response by wall motion pattern and lead location

	All	Non-T2CL	CL Only	T2 Only	T2CL	*P* value^a^
	*N* = 33	*N* = 21 (64 %)	*N* = 18 (55 %)	*N* = 20 (61 %)	*N* = 12 (36 %)	
∆ESV (ml)	−24 ± 40	−19 ± 48	−31 ± 40^b^	−30 ± 24^c^	−33 ± 18	0.023
Echocardiographic responder^d^, n (%)	18 (55)	7 (33)	13 (72)	14 (70)	11 (92)	0.003

^a^The *p* values listed in the table are for the comparison of T2CL versus non-T2CL (*type I* wall motion pattern and/or a remote left ventricular lead)

^b^For a remote lead, regardless of wall motion pattern, ∆ESV was −15 ± 40 ml. Compared to those with CL, *p* = 0.11

^c^For a type I wall motion pattern, regardless of lead concordance, ∆ESV −15 ± 56 ml. Compared to those with T2, *p* = 0.019

^d^Echocardiographic response was defined by a decrease in ESV by ≥ 15 % at 6 month follow-up

CL = Concordant left ventricular lead; ESV = End-Systolic Volume; T2 = *Type II* wall motion pattern; T2CL = *Type II* wall motion pattern and a concordant left ventricular lead

### Response: ECG patterns

The response rate for patients with favorable ECGs was 69 %, compared to 45 % for those with non-favorable ECGs (*p* = 0.28). In those with non-favorable ECGs, the presence of T2CL was associated with significantly greater likelihood of response compared to those without T2CL (86 % versus 23 %, *p* = 0.017).

### Response: LGE and NICM

Thirty patients (91 %) underwent LGE imaging (3 were excluded due to severe renal insufficiency or patient discomfort necessitating early exam termination) and 8 patients (24 %) had significant scar (6 with a *type I* pattern and 2 with a *type II* pattern). The response rate in patients with significant scar was 25 %, compared to 64 % for those without scar (*p* = 0.10). Four patients had an LV lead located in a non-viable segment, with a 25 % response rate in this group. The low prevalence of significant LGE precluded statistical analysis of the effect of T2CL in patients with scar; however, in the subgroup without significant scar, the presence of T2CL was associated with significantly greater response compared to non-T2CL (92 % versus 39 %, *p* = 0.011).

### Predictive modeling and incremental benefit of CMR wall motion pattern type and lead concordance

On univariate analysis, NICM (odds ratio [OR] 11, 95 % confidence interval [CI] 1.2-109, *p* = 0.036), a *type II* pattern (OR 5.3, 95 % CI 1.2-24, *p* = 0.032), concordant LV lead location (OR 5.2, 95 % CI 1.2-23, *p* = 0.030), and T2CL (OR 22, 95 % CI 2.3-206, *p* = 0.007) were the only significant predictors of response. Variables included in multivariate analysis are shown in Table 4. T2CL was the only *independent* predictor of response. The addition of T2CL to NICM and a favorable ECG resulted in an increase in AUC for response from 0.69 to 0.84 (*p* = 0.038). The presence of significant scar was not evaluated in these primary analyses due to the low prevalence of significant LGE as well as an incomplete data set for this parameter for the three patients who did not undergo LGE imaging. Intermediary analyses with incremental addition of each covariate into the model along with T2CL are also shown; results did not deviate significantly between these intermediary models and the final model (Table 5).

**Table 4:** Predictors of echocardiographic response

	Univariate			Multivariate		
Variable	Odds ratio	95 % confidence interval	*P* value for logistic regression	Odds ratio	95 % confidence interval	*P* value for logistic regression^a^
NICM	11	1.2-109	0.036	3.1	0.3-37	0.36
Favorable ECG^b^	2.8	0.6-12	0.18	3.0	0.5-19	0.25
T2CL	22	2.3-206	0.007	18	1.6-206	0.018

^a^No significant difference with 1000 sample bootstrapped multivariate logistic regression analysis

(*p* = 0.012 for T2CL, *p* = non-significant for all others)

^b^A favorable ECG pattern is indicated by the presence of a true left bundle branch block and a QRS duration > 150 ms

ECG = Electrocardiogram; NICM = Non-Ischemic Cardiomyopathy; T2CL = *Type II* wall motion pattern and a concordant left ventricular lead

**Table 5:** Intermediary multivariate models

	Model 1 – T2CL and NICM only			Model 2 – T2CL and Favorable ECG only		
Variable	Odds ratio	95 % confidence interval	*P* value for logistic regression	Odds ratio	95 % confidence interval	*P* value for logistic regression^a^
NICM	4.5	0.4-48	0.21	--	--	--
Favorable ECG^a^	--	--	--	3.9	0.6-23.7	0.14
T2CL	15	1.5-147	0.02	27	2.6-276	0.006

^a^A favorable ECG pattern is indicated by the presence of a true left bundle branch block and a QRS duration > 150 ms

ECG = Electrocardiogram; NICM = Non-Ischemic Cardiomyopathy; T2CL = *Type II* wall motion pattern and a concordant left ventricular lead

### Exploratory predictive model

The exploratory addition of maximum opposing wall delay, presence of septal flash, and presence of significant scar to the multivariate model did not significantly change results, with T2CL remaining the only independent predictor of response (OR 25, 95 % CI 1.4-453, *p* = 0.030; 1000 sample bootstrapping: *p* = 0.006 for T2CL and *p* = non-significant for all others); with an increase in AUC for response from 0.71 to 0.86.

### Reproducibility of wall motion patterns and lead position

Two independent observers (GH and JO) reviewed all 33 regional wall motion maps and categorized each as either *type I* or *type II*, demonstrating substantial agreement (88 %) with a Cohen’s kappa coefficient of 0.75 (*p* < 0.0001). Two independent observers (GH and ML) reviewed 15 randomly selected biplane fluoroscopic images to assess if the LV lead was concordant or remote to the latest site, demonstrating excellent agreement (93 %) with a Cohen’s kappa coefficient of 0.87 (*p* = 0.001). In both cases, discrepancies were resolved by consensus review of the two observers to determine the final classification.

## Discussion

We demonstrated that the combination of a *type II* left ventricular wall motion pattern and localization of a pacing lead concordant with the latest contracting LV site independently predicted echocardiographic response to CRT. It is likely that previously identified clinical predictors of response, such as NICM and a favorable ECG pattern are useful due to the relatively high proportion of *type II* patterns present in these groups. In addition to supporting this trend, our study showed that *type II* patterns are also present in other less favorable groups, for example, nearly half (45 %) of those with non-favorable ECG. To date, imaging of dyssynchrony has largely been regarded generically as a delay in mechanical contraction between the septal and lateral walls, and prospective utilization of these criteria for CRT selection has yielded disappointing results [2]. Recent refinements in imaging methods aimed at improving CRT response have focused on identification of specific patterns of LV wall motion or targeting the site of latest LV contraction [8, 9, 11, 12]. The present study is the first report on the interaction of lead location and specific wall motion patterns.

Dichotomous dyssynchrony patterns were first identified on invasive electrophysiologic mapping of endocardial electrical activation in patients with left intraventricular conduction delays and classified as *type I* and *type II* [22–24]. The two patterns were generally distinguished by: 1) the degree of trans-septal electrical delay and 2) the pattern of LV activation relative to the presence or absence of local conduction block. The *type I* pattern is characterized by short trans-septal delay and homogenous slow LV activation towards the lateral wall, likely related to electromechanical uncoupling from myopathy instead of specific conduction system disease (i.e. of the left bundle branch). The *type II* pattern is characterized by prolonged trans-septal delay and an inferoseptal or anteroseptal breakout site with a corresponding functional line of block resulting in a “u” or “n” shaped LV activation pattern indicative of a primary electrical abnormality. “Correction” of the *type II* pattern with CRT is associated with mid-term clinical and echocardiographic improvement, while many patients with *type I* patterns fail to significantly respond [7, 15]. Fung et al. demonstrated that *type II* patients identified by invasive electrophysiologic mapping had overall improvement in echocardiographic dyssynchrony after CRT, while *type I* patients actually had worsening dyssynchrony [7]. Thus, a *type II* pattern likely represents a discrete line of conduction block more amenable to electrical resynchronization compared to the diffuse, homogenous conduction delay of *type I*.

The discussion above linking mechanical contraction patterns and electrical conduction patterns depends on the amount of electro-mechanical (EM) coupling that is present. We previously conducted a study in patients undergoing CRT who had a pre-procedural CMR exam. During the CRT procedure, local intracardiac electrograms were taken at various sites, and electric times from the electrograms were correlated to mechanical contraction times from the CMR studies. We found that electrical and mechanical delay times were highly correlated in individual patients, indicating a high level of EM coupling [13].

Recently, Sohal et al. used CMR to define *type I* and *type II* patterns of wall motion in 52 patients planning to undergo CRT [12]. Utilizing an endocardial displacement analysis similar to ours, they established the *type II* pattern as the only independent predictor of echocardiographic response at 6 months. Similarly, we demonstrated a higher likelihood of response in *type II* patients alone (70 %, versus 31 % for *type I*), however, the likelihood of response was far superior in *type II* patients with a concordant LV lead (92 %). Although the *type II* pattern is more common in NICM, which is a dependable predictor of CRT response [25, 26], both our study and that of Sohal et al. included NICM in multivariate analyses without a change in the final results. We found the *type I* pattern to be more common in patients with ischemic cardiomyopathy (ICM) and significant scar, however, only a small portion of our patients had ICM, precluding a subgroup analysis of the effects of T2CL within this group. In the study by Sohal et al., 48 % of the patients had ICM and *type II* patients with ICM had more than double the response rate of *type I* patients [12]. The relationship between a *type II* pattern and response to CRT persisted after correcting for significant LV scar. Furthermore, another recent study has suggested that the presence of a septal flash is an important predictor of CRT response in addition to scar absence [27]. Our exploratory analysis indicates that T2CL predicts CRT response after correction for both scar presence and septal flash, although limitations of modeling preclude strong conclusions.

Conventional CRT implantation methods targeting the most anatomically suitable lateral or posterior coronary vein may contribute to poor response. Often, the latest contracting LV segment is not located posteriorly or laterally [14]. Furthermore, implantation of the LV lead into a segment with significant scar is undesirable [16, 17]. A small study utilizing CMR to target the latest contracting segment without significant scar demonstrated superior echocardiographic response in those with targeted leads compared to those without [10]. Larger randomized controlled trials using speckle-tracking echocardiography to guide LV lead placement to the latest contracting LV site have also demonstrated superior clinical and echocardiographic responses for those in the target arms versus the control arms [8, 9]. Our findings expand upon these previous studies and suggest that targeting a particular substrate (the *type II* pattern) along with a particular site (the latest to contract) may yield the highest likelihood of response.

Currently, the ECG characteristics of true LBBB morphology with a QRS duration > 150 ms are considered the most important predictors of CRT response [4–6]. Contemporary dyssynchrony imaging may be complementary to ECG findings [28], and patients with less favorable ECG characteristics may stand to gain the most from imaging to determine CRT candidacy and a targeted LV lead approach [29]. In our study, T2CL was a significantly associated with CRT response in patients without favorable ECG findings. It has been suggested that patients with a QRS duration < 120–130 ms should not undergo CRT, regardless of the presence of dyssynchrony [30] and patients without dyssynchrony on imaging may actually be harmed by CRT [31]. Therefore, a focus on imaging to refine candidacy and facilitate optimized interventions may be more appropriate than ongoing efforts to expand CRT candidacy. Although T2CL was present in a minority of patients (36 %), the *type II* pattern was present in the majority (61 %) and may represent an opportunity to improve response by targeting this substrate.

### Limitations

This is a small study, which increases the likelihood of results occurring due to chance. An additional 1000 sample bootstrapping analysis was performed to ensure internal validity. Our method utilizes radial displacement of LV myocardium to characterize regional wall motion, which does not directly measure mechanical contraction onset or peaks. Although translational movement is corrected, passive motion cannot be totally excluded; however, our method has been validated against electrical activation patterns obtained by regional intra-operative sampling [13]. The degree of processing time required for manual correction of automated edge detection is another limitation of the technique, which is approximately 2 hours per case. We are currently pursuing and evaluating methods to increase the speed of processing. Reduction of ESV is a surrogate imaging end-point [32], however, echocardiographic response has been found to be a superior predictor of intermediate and long-term hard outcomes in CRT patients compared to clinical response [33, 34]. The observational nature of our study did not allow the investigation of targeting of lead placement based on CMR findings. As such, our study does not prove that actively targeting the latest site in *type II* patients provides superior response; we simply demonstrated the independent association of these features with a higher likelihood of response. A prospective randomized interventional trial of CRT guided by CMR derived wall motion patterns in a larger group of patients would be the next step in the validation of this approach.

## Conclusion

The combination of a *type II* U-shaped LV wall motion pattern identified by CMR and concordant LV lead position predicts the highest likelihood of response among patients who meet current CRT implantation criteria. Improving CRT response rates may ultimately rely on patient selection by wall motion pattern and targeted LV lead placement, both of which are facilitated by CMR.

## Abbreviations

AUC: Area under the receiver-operating curve
CI: Confidence interval
CMR: Cardiovascular magnetic resonance
CRT: Cardiac resynchronization therapy
ECG: Electrocardiogram
EF: Ejection fraction
EM: Electro-mechanical
ESV: End-systolic volume
ICM: Ischemic cardiomyopathy
LBBB: Left bundle branch block
LGE: Late gadolinium enhancement
LV: Left ventricular
NICM: Non-ischemic cardiomyopathy
OR: Odds ratio
T2CL: *Type II* wall motion pattern with concordant left ventricular lead placement

## References

[1] Versteeg H, Schiffer AA, Widdershoven JW, Meine MM, Doevendans PA, Pedersen SS (2009). Response to cardiac resynchronization therapy: is it time to expand the criteria?. Pacing Clin Electrophysiol.

[2] Chung ES, Leon AR, Tavazzi L, Sun JP, Nihoyannopoulos P, Merlino J (2008). Results of the Predictors of Response to CRT (PROSPECT) trial. Circulation.

[3] Russo AM, Stainback RF, Bailey SR, Epstein AE, Heidenreich PA, Jessup M (2013). ACCF/HRS/AHA/ASE/HFSA/SCAI/SCCT/SCMR 2013 appropriate use criteria for implantable cardioverter-defibrillators and cardiac resynchronization therapy: a report of the American College of Cardiology Foundation appropriate use criteria task force, Heart Rhythm Society, American Heart Association, American Society of Echocardiography, Heart Failure Society of America, Society for Cardiovascular Angiography and Interventions, Society of Cardiovascular Computed Tomography, and Society for Cardiovascular Magnetic Resonance. J Am Coll Cardiol.

[4] Dupont M, Rickard J, Baranowski B, Varma N, Dresing T, Gabi A (2012). Differential response to cardiac resynchronization therapy and clinical outcomes according to QRS morphology and QRS duration. J Am Coll Cardiol.

[5] Sipahi I, Carrigan TP, Rowland DY, Stambler BS, Fang JC (2011). Impact of QRS duration on clinical event reduction with cardiac resynchronization therapy: meta-analysis of randomized controlled trials. Arch Intern Med.

[6] Stavrakis S, Lazzara R, Thadani U (2012). The benefit of cardiac resynchronization therapy and QRS duration: a meta-analysis. J Cardiovasc Electrophysiol.

[7] Fung JW, Chan JY, Yip GW, Chan HC, Chan WW, Zhang Q (2007). Effect of left ventricular endocardial activation pattern on echocardiographic and clinical response to cardiac resynchronization therapy. Heart.

[8] Khan FZ, Virdee MS, Palmer CR, Pugh PJ, O'Halloran D, Elsik M (2012). Targeted left ventricular lead placement to guide cardiac resynchronization therapy: the TARGET study: a randomized, controlled trial. J Am Coll Cardiol.

[9] Saba S, Marek J, Schwartzman D, Jain S, Adelstein E, White P (2013). Echocardiography-guided left ventricular lead placement for cardiac resynchronization therapy: results of the Speckle Tracking Assisted Resynchronization Therapy for Electrode Region trial. Circ Heart Fail.

[10] Shetty AK, Duckett SG, Ginks MR, Ma Y, Sohal M, Bostock J (2013). Cardiac magnetic resonance-derived anatomy, scar, and dyssynchrony fused with fluoroscopy to guide LV lead placement in cardiac resynchronization therapy: a comparison with acute haemodynamic measures and echocardiographic reverse remodelling. Eur Heart J Cardiovasc Imaging.

[11] Bilchick KC, Kuruvilla S, Hamirani YS, Ramachandran R, Clarke SA, Parker KM (2014). Imapct of mechanical activation, scar, and electrical timing on cardiac resynchronization therapy response and clinical outcomes. J Am Coll Cardiol.

[12] Sohal M, Shetty A, Duckett S, Chen Z, Sammut E, Amraoui S (2013). Noninvasive assessment of LV contraction patterns using CMR to identify responders to CRT. JACC Cardiovasc Imaging.

[13] Suever JD, Hartlage GR, Magrath RP, Iravanian S, Lloyd MS, Oshinski JN (2014). Relationship between mechanical dyssynchrony and intra-operative electrical delay times in patients undergoing cardiac resynchronization therapy. J Cardiovasc Magn Reson.

[14] van Bommel RJ, Ypenburg C, Mollema SA, Borleffs CJ, Delgado V, Bertini M (2011). Site of latest activation in patients eligible for cardiac resynchronization therapy: patterns of dyssynchrony among different QRS configurations and impact of heart failure etiology. Am Heart J.

[15] Duckett SG, Camara O, Ginks MR, Bostock J, Chinchapatnam P, Sermesant M (2012). Relationship between endocardial activation sequences defined by high-density mapping to early septal contraction (septal flash) in patients with left bundle branch block undergoing cardiac resynchronization therapy. Europace.

[16] Bilchick KC, Dimaano V, Wu KC, Helm RH, Weiss RG, Lima JA (2008). Cardiac magnetic resonance assessment of dyssynchrony and myocardial scar predicts function class improvement following cardiac resynchronization therapy. JACC Cardiovasc Imaging.

[17] Petryka J, Misko J, Przybylski A, Spiewak M, Malek LA, Werys K (2012). Magnetic resonance imaging assessment of intraventricular dyssynchrony and delayed enhancement as predictors of response to cardiac resynchronization therapy in patients with heart failure of ischaemic and non-ischaemic etiologies. Eur J Radiol.

[18] Fantoni C, Kawabata M, Massaro R, Regoli F, Raffa S, Arora V (2005). Right and left ventricular activation sequence in patients with heart failure and right bundle branch block: a detailed analysis using three-dimensional non-fluoroscopic electroanatomic mapping system. J Cardiovasc Electrophysiol.

[19] White JA, Yee R, Yuan X, Krahn A, Skanes A, Parker M (2006). Delayed enhancement magnetic resonance imaging predicts response to cardiac resynchronization therapy in patietns with intraventricular dyssynchrony. J Am Coll Cardiol.

[20] Suever JD, Fornwalt BK, Neuman LR, Delfino JG, Lloyd MS, Oshinski JN (2014). Method to create regional mechanical dyssynchrony maps from short-axis cine steady-state free-precession images. J Magn Reson Imaging.

[21] Cerqueira MD, Weissman NJ, Dilsizian V, Jacobs AK, Kaul S, Laskey WK (2002). Standardized myocardial segmentation and nomenclature for tomographic imaging of the heart. A statement for healthcare professionals from the Cardiac Imaging Committee of the Council on Clinical Cardiology of the American Heart Association. Circulation.

[22] Auricchio A, Fantoni C, Regoli F, Carbucicchio C, Goette A, Geller C (2004). Characterization of left ventricular activation in patients with heart failure and left bundle-branch block. Circulation.

[23] Fung JW, Yu CM, Yip G, Zhang Y, Chan H, Kum CC (2004). Variable left ventricular activation pattern in patients with heart failure and left bundle branch block. Heart.

[24] Rodriguez LM, Timmermans C, Nabar A, Beatty G, Wellens HJ (2003). Variable patterns of septal activation in patients with left bundle branch block and heart failure. J Cardiovasc Electrophysiol.

[25] Goldenberg I, Moss AJ, Hall WJ, Foster E, Goldberger JJ, Santucci P (2011). Predictors of response to cardiac resynchronization therapy in the Multicenter Automatic Defibrillator Implantation Trial with Cardiac Resynchronization Therapy (MADIT-CRT). Circulation.

[26] McLeod CJ, Shen WK, Rea RF, Friedman PA, Hayes DL, Wokhlu A (2011). Differential outcome of cardiac resynchronization therapy in ischemic cardiomyopathy and idiopathic dilated cardiomyopathy. Heart Rhythm.

[27] Sohal M, Amraoui S, Chen Z, Sammut E, Jackson T, Wright M (2014). Combined identification of septal flash and absence of myocardial scar by cardiac magnetic resonance imaging improves prediction of response to cardiac resynchronization therapy. J Interv Card Electrophysiol.

[28] Hara H, Oyenuga OA, Tanaka H, Adelstein EC, Onishi T, McNamara DM (2012). The relationship of QRS morphology and mechanical dyssynchrony to long-term outcome following cardiac resynchronization therapy. Eur Heart J.

[29] Marek JJ, Saba S, Onishi T, Ryo K, Schwartzman D, Adelstein EC (2014). Usefulness of Echocardiographically Guided Left Ventricular Lead Placement for Cardiac Resynchronization Therapy in Patients With Intermediate QRS Width and Non-Left Bundle Branch Block Morphology. Am J Cardiol.

[30] Ruschitzka F, Abraham WT, Singh JP, Bax JJ, Borer JS, Brugada J (2013). Holzmeister J; EchoCRT Study Group. Cardiac-resynchronization therapy in heart failure with a narrow QRS complex. N Engl J Med.

[31] Auger D, Bleeker GB, Bertini M, Ewe SH, van Bommel RJ, Witkowski TG (2012). Effect of cardiac resynchronization therapy in patients without left intraventricular dyssynchrony. Eur Heart J.

[32] Fornwalt BK, Sprague WW, BeDell P, Suever JD, Gerritse B, Merlino JD (2010). Agreement is poor among current criteria used to define response to cardiac resynchronization therapy. Circulation.

[33] Ypenburg C, van Bommel RJ, Borleffs CJ, Bleeker GB, Boersma E, Schalij MJ (2009). Long-term prognosis after cardiac resynchronization therapy is related to the extent of left ventricular reverse remodeling at midterm follow-up. J Am Coll Cardiol.

[34] Yu CM, Bleeker GB, Fung JW, Schalij MJ, Zhang Q, van der Wall EE (2005). Left ventricular reverse remodeling but not clinical improvement predicts long-term survival after cardiac resynchronization therapy. Circulation.

